# Surveying Nutrient Assessment with Photographs of Meals (SNAPMe): A Benchmark Dataset of Food Photos for Dietary Assessment

**DOI:** 10.3390/nu15234972

**Published:** 2023-11-30

**Authors:** Jules A. Larke, Elizabeth L. Chin, Yasmine Y. Bouzid, Tu Nguyen, Yael Vainberg, Dong Hee Lee, Hamed Pirsiavash, Jennifer T. Smilowitz, Danielle G. Lemay

**Affiliations:** 1 United States Department of Agriculture, Agricultural Research Service, Western Human Nutrition Research Center, Davis, CA 95616, USA; 2 Department of Nutrition, University of California Davis, Davis, CA 95616, USA; 3 Department of Computer Science, University of California Davis, Davis, CA 95616, USAhpirsiav@ucdavis.edu (H.P.)

**Keywords:** food photos, dietary assessment, food records, artificial intelligence, computer vision

## Abstract

Photo-based dietary assessment is becoming more feasible as artificial intelligence methods improve. However, advancement of these methods for dietary assessment in research settings has been hindered by the lack of an appropriate dataset against which to benchmark algorithm performance. We conducted the Surveying Nutrient Assessment with Photographs of Meals (SNAPMe) study (ClinicalTrials ID: NCT05008653) to pair meal photographs with traditional food records. Participants were recruited nationally, and 110 enrollment meetings were completed via web-based video conferencing. Participants uploaded and annotated their meal photos using a mobile phone app called Bitesnap and completed food records using the Automated Self-Administered 24-h Dietary Assessment Tool (ASA24^®^) version 2020. Participants included photos before and after eating non-packaged and multi-serving packaged meals, as well as photos of the front and ingredient labels for single-serving packaged foods. The SNAPMe Database (DB) contains 3311 unique food photos linked with 275 ASA24 food records from 95 participants who photographed all foods consumed and recorded food records in parallel for up to 3 study days each. The use of the SNAPMe DB to evaluate ingredient prediction demonstrated that the publicly available algorithms FB Inverse Cooking and Im2Recipe performed poorly, especially for single-ingredient foods and beverages. Correlations between nutrient estimates common to the Bitesnap and ASA24 dietary assessment tools indicated a range in predictive capacity across nutrients (cholesterol, adjusted R^2^ = 0.85, *p* < 0.0001; food folate, adjusted R^2^ = 0.21, *p* < 0.05). SNAPMe DB is a publicly available benchmark for photo-based dietary assessment in nutrition research. Its demonstrated utility suggested areas of needed improvement, especially the prediction of single-ingredient foods and beverages.

## 1. Introduction

Collecting dietary intake data is an essential part of human nutrition research. However, conventional methods such as food frequency questionnaires (FFQs), 24 h dietary recalls, and food records are time-consuming, burdensome to participants, and are prone to biases and errors [1,2]. FFQs and dietary recalls rely on participant memory to adequately describe and to recall their diet during the study period. The conventional methods also have an inherent bias related to self-reporting [3].

A growing body of work is aimed towards recognizing and classifying foods from photos. These methods use computer vision, which is a field of artificial intelligence that makes images intelligible to computers so that information can be extracted. Convolutional neural networks have been used to classify foods from a variety of cultures [4,5,6,7]. Some work has also been carried out to estimate portion size, an essential step for nutrient estimation. Researchers developed an algorithm to estimate energy from food images, which showed promising results for meals less than 1000 Kcal [8]. Im2Calories identifies foods, estimates the size, and predicts the calories from the nutrition information [9]. Some commercial phone apps, such as Bitesnap (Bite AI, New York, NY, USA), Calorie Mama (Azumio, Palo Alto, CA, USA), and MyFitnessPal (Francisco Partners, San Francisco, CA, USA) also predict food and nutrition information from food photos taken from mobile phones. There has also been research to predict ingredients from food images with the primary goal of recipe prediction [10,11,12]. Ingredient prediction is especially desirable for human nutrition research because it allows for the estimation of specific food components or linking to other food and nutrition databases [13]. Methods for the prediction of foods and their volumes from photos have been reviewed extensively elsewhere [14,15] and continue to evolve rapidly. A key limitation in the field of image-based dietary assessment is the inability to compare different image-processing methods on the same food photos collected in the context of dietary assessment.

One of the earliest mobile food record systems developed for research purposes was the Technology Assisted Dietary Assessment (TADA) at Purdue University in 2010 [16,17], with later improvements for portion size and food energy estimation [8]. The energy estimates of the mobile food record (mFR) have been validated with doubly labeled water, with accuracy comparable to traditional food records [18]. Another pioneering method was the Remote Food Photography Method, which was developed as a smartphone app called SmartIntake [19]. In 2023, researchers reported a top-five food classification accuracy of 97.6% and food volume estimation of 10% on an image dataset of Mediterranean foods [20]. Many other mobile food record applications, such as MyFitnessPal [21], Eat and Track [22], FoodNow [23], EVIDENT [24], e-DIA [25,26], and Keenoa [27,28], have been developed for image-based dietary assessment. A meta-analysis of validation studies performed on dietary record applications found that such apps underestimate energy and macronutrients relative to traditional methods [29]. Comparisons of micronutrient and food group consumption could not be made on account of non-comparable data. How do photo-based methods compare to each other on the task of dietary intake assessment? A common benchmark that includes both food photos and accompanying diet records mapped to food composition databases is needed.

Although many food image datasets are available with multiple food categories and types, including Food-101, VireoFood-172, UEC FOOD-256, and MedGRFood, among others [20,30,31,32,33,34,35,36], few food image datasets include ingredient labels [10,11,37]. Most recently, using photos mined from the web, a database was developed to map food images to a USDA Food Composition database [38]. However, Internet images may be staged or not reflective of “real-life” eating. To our knowledge, there is no publicly available food image dataset derived from camera-phone photos taken by free-living humans in the context of dietary assessment and which is fully labeled with food text descriptions and food codes.

The purpose of the Surveying Nutrient Assessment with Photographs of Meals (SNAPMe) study was to build an image database of food photos taken by free-living human participants using phone cameras. This database may be used to evaluate artificial intelligence algorithms for mobile phone image-based dietary assessment. Bitesnap was selected as the phone app for the ease of researcher access the backend (the photos) via an API. However, Bitesnap was used only to capture photos, not for ground truth. As a ground truth for the photos, knowledgeable and trained participants entered food records using the Automated Self-Administered 24-h (ASA24) Dietary Assessment Tool version 2020 [39]. The food photos and food records were extensively inspected for correctness, with each line of each food record linked to the appropriate food photo. The collection was uploaded as the SNAPMe DB and is now publicly available on Ag Data Commons [40]. Existing A.I. algorithms were evaluated using the SNAPMe benchmark to demonstrate its utility and to identify weaknesses of current methods.

## 2. Materials and Methods

### 2.1. Participants

The SNAPMe study was a completely remote study that did not require in-person interaction between study coordinators/staff and participants. Participants were recruited starting in July of 2021. Study advertisements were emailed to nutrition and food science programs throughout the U.S., previous UC Davis/Foods for Health Institute study participants, health and wellness clinics in the greater Sacramento, California area, and community/cultural centers in the Northern California area; flyers were also posted in public areas throughout the Sacramento, CA area.

Interested individuals were invited to take a screening survey; those who met the following eligibility criteria were invited to participate in the study: Participants were to be generally weight-stable, healthy adults aged 18–65 years; consume at least three meals per day; consume at least 50% of meals at home vs. outside the home (e.g., dine-in restaurants, take-out, etc., because the ingredients would be unknown); prepare at least 50% of the prepared meals in their household; be willing to consume a variety of food throughout the study; refrain from consuming meal substitution items (e.g., meal replacement bars or shakes); currently own a smartphone with a working camera and to have owned and used a smartphone for the last 12 months; download an app linked to their smartphone, camera, and phone storage; spend 1–2 h per day recording their meals on each study day; refrain from sharing meals with other participants during data collection; refrain from participating in other studies and elective surgeries during the study period; and pass a food-matching test in the online screening survey with a score >70% (described below). Individuals were excluded if any of the following exclusion criteria were met prior to study enrollment: pregnant or planning on becoming pregnant during the study period, having a history of any type of eating disorder, or currently using restrictive diets (e.g., caloric restriction or intermittent fasting). Using a phone with the Android operating system v10 was added as an exclusion criterium midway through the study because of incompatibility issues with the app; however, using earlier or later versions of Android OS was an acceptable criterium.

The purpose of the food matching test was to assess whether participants could adequately identify and describe foods from photos. The food matching test consisted of ten questions; eight questions asked participants to identify the food(s) shown in photos of mixed dishes (i.e., contained multiple ingredients or foods), and two questions asked them to estimate the serving size of the food in the photo. All questions were multiple choice. Each question was first scored individually, with a “pass” defined as selecting at least *n* correct choices and fewer than *k* choices, and a “no pass” defined as selecting more than k choices and fewer than *n* correct choices, where *n* was typically 75% of the correct answers and *k* was typically 67% of the total number of options available (i.e., participants could not select all or almost all of the answers and get a “pass”). An example of a screening food matching question is in Supplemental Figure S1. The overall score was considered a “pass” if at least 7 of the 10 questions had a “pass”. A total of 279 individuals took the screening questionnaire and 196 individuals passed.

Individuals who passed the screening survey were contacted to confirm their study interest. Those willing to participate in the study were mailed study materials that included study instructions, sizing markers for use with recording food photos, and a study checklist. After receiving study materials, participants were scheduled for a virtual enrollment visit where study coordinators confirmed that participants met the inclusion criteria, taught participants how to download Bitesnap to their smartphone, and how to record their diet through Bitesnap and ASA24. A total of 110 participants were enrolled. Participants were withdrawn if they were unresponsive (*n* = 11), if they were unable to complete the study in the allotted time (*n* = 3), and if they were using a phone with Android operating system v10 (*n* = 3) or dissatisfied with the team response (*n* = 1).

### 2.2. Questionnaires

A Health History Questionnaire (HHQ) was administered to enrolled participants in an online survey format (Qualtrics). The HHQ collected information about participant demographics, phone models, general health, supplement intake, and diet. Dietary questions pertained to food preparation and consumption habits, adherence to special diets, the frequency of food/ingredient substitutions or exclusions, the type of cultural cuisine typically consumed, and the consumption of types of foods that might be difficult to visually distinguish (e.g., lactose-free dairy products vs. regular dairy products).

At the end of the study, participants were administered a post-study questionnaire in an online survey format (Qualtrics). Participants were asked to rate statements regarding ASA24 and Bitesnap for the ability to capture diet accurately, the ease of finding foods, and overall burden on a seven-point scale (“strongly disagree” to “strongly agree”), in addition to when they typically logged their diet with respect to when they ate. Participants were also given the opportunity to write comments about what they liked and disliked about recording diet, foods that were difficult to report, and whether they preferred either of the dietary data recording methods.

### 2.3. Data Recording

Participants were asked to report three days of dietary intake through food records using ASA24-2020 software and through a mobile phone app called Bitesnap. To ensure a variety of foods/meals were captured, participants were asked to collect data on two weekdays and one weekend day. Participants were instructed to record all food and beverages, excluding gum, seasonings, and water, as soon as possible after eating, with all data required to be entered by midnight of the study day. When a participant ate something where they were unsure of the ingredients, they were advised to find a similar recipe online to guide entering their data into Bitesnap or ASA24. Participants were compensated up to $100 to complete all three days of dietary data recording. Data collection ended in January 2022.

### 2.4. Recording Diet in Bitesnap

Participants were asked to take “Before” and “After” eating photos of non-packaged meals, foods, and beverages, or the package label front (“Package Front”) and “Ingredient Label” photos of single-serving packaged foods to upload to Bitesnap. Multiple-serving packaged food and drinks, defined as those where the participant intended to eat only part of the package contents, were treated as “non-packaged”, and participants were asked to take “Before” and “After” photos of the food/drinks as served in their bowl/plate/cup. For non-packaged foods, participants were asked to place a sizing marker on the bottom left of their meal to assist with size estimation. The sizing marker was a black and white checkerboard pattern where each individual square was 0.75″ × 0.75″ (for a total length × width of 1.5″ × 1.5″). Participants were asked to take photos at a distance of approximately 18” and at a 45-degree angle. For packaged food (“Package Front” and “Ingredient Label”) photos, participants were asked to take the photo head-on and close enough to make text legible; the sizing marker did not need to be included with the packaged food photos. Participants were instructed to take photos of each plate/bowl/cup separately and include all the food in a single plate/bowl. The serving size was the amount in the photo, not the amount the participants consumed.

Participants were instructed to label “Before” and “Package Front” photos either using the suggested labels or to enter their own label and to add food details when prompted by the app. Additional details and information about each entry could be freely written in by participants as separate notes. To avoid participants having to completely annotate and label corresponding “After” and “Ingredient Label” photos with each food/beverage and details, participants labeled those photos using custom labels for “After” and “Ingredient Label”. The custom “After” and “Ingredient Label” labels were assigned 0s for all nutrient values and the serving size was always set to 1 (with no unit). When participants ate another helping of a food/meal, they were instructed to upload unique “Before” and “After” photos corresponding to the additional helping; to avoid participants having to annotate and label the entries a second time, they were asked to label the “Before” photo using a custom label called “Another Helping” and the “After” photo using the “After” label. The “Another Helping” label had a serving size of 1 (no unit) and 0s for all nutrient values.

Participants emailed Bitesnap data to the study coordinators at the end of every study day. Study personnel checked for data completeness and downloaded all photos within 48 h of receipt. Participants were asked to revise their Bitesnap data to upload missing entries (e.g., missing “After” images) or change labels if needed (e.g., using “After” instead of “Ingredient Label”). Bitesnap automatically crops photos to 800 × 800 pixels; participants were asked to send the original (uncropped) versions of photos when parts of the food, beverage, or sizing marker were cut off during the automated cropping step. Data with photos missing sizing markers in approximately one-third of photos or approximately one-third of entries without corresponding “After” or “Ingredient Label” photos were considered incomplete, and participants were given the option to do a make-up day that involved recording diet in both ASA24 and Bitesnap on another day.

### 2.5. Recording Diet in ASA24

Participants were instructed to enter their diet using the ASA24 system in food record mode. For foods that were not in the ASA24 system, participants were encouraged to use the “Add a recipe” function to construct the food from ingredients. The ASA24RecallTracking file was used to assure that participants recorded at least 3 meals. When participants recorded too few meals, participants were given the option to do a make-up day.

### 2.6. Data Quality Control (QC)

After study completion, data were reviewed and organized to link the ASA24 entries file with Bitesnap entries using a uniform standard operating procedure. The purpose of data QC was to link the ASA24 and Bitesnap data. For entries that appeared to be missing based on photos, data were not altered by study personnel to add or change entries in ASA24 or Bitesnap. Instead, ASA24 entries that could not be mapped to a food photo were listed in an “NAlist.txt” file in the SNAPMe database so that they could be incorporated into an analysis, depending on the purpose of the analysis.

For the Bitesnap QC, the photo filename was checked for each entry. Multiple entries may have corresponded to the same photo if multiple foods were pictured in a single photo. Filenames are a string of 28 random letters and numbers. Photos are in JPEG format. “After” photos have the same filename nomenclature as {before_filename} appended with “_after”, where {before_filename} is the filename for the corresponding “Before” photo. Similarly, “Ingredient Label” photos have the filename nomenclature as {packagefront_filename} appended with “_ingredients”, where {packagefront_filename} is the filename of the corresponding “Package Front” photo.

When the photo file was unavailable, for example, if the file could not be downloaded because of a glitch in the Bitesnap output, but the participant sent the original photo, then the original photo was used, a random filename was generated, and the nomenclature described above was used. Entries with missing photos but with text labels, or entries missing altogether (e.g., there was a “Before” entry but no “After”) were logged in the missing photos file. Entries corresponding to “Another Helping” and their corresponding “After” photos were removed from the dataset.

The ASA24 Items file, which was one of the researcher files produced by the ASA24 system, was used to obtain the individual food entries for each study day. The ASA24 entries were cross-checked with the Bitesnap entries to link each Bitesnap photo with the corresponding ASA24 entries. The ASA24 FoodNum, which corresponded to the sequence that the food was reported, was used to help link ASA24 and Bitesnap meals that had different numbers of individual entries per meal. For example, a food may have been reported as “Sandwich” in Bitesnap (one row), but the individual ingredients/foods that comprised the sandwich (“bread”, “cheese”, “lettuce”, etc.) were output in ASA24 (multiple rows), all of which had the same FoodNum. In ASA24, when multiple foods with different FoodNums corresponded to the same meal/entry (e.g., “half and half” was reported as a separate food instead of as an addition to “coffee”, so it was given a FoodNum different from “coffee”), the lowest FoodNum corresponding to the entire meal/entry was used for the corresponding Bitesnap meal/entry.

### 2.7. Statistics

To compare predicted scores with the ground truth data, F1 scores were calculated. F1 scores, the harmonic mean of precision and recall, were calculated using the set of tokens (unique individual words) from the ASA24 description and the predicted set based on each food photo. True positives were counted as tokens correctly predicted by the algorithm (FB Inverse Cooking or Im2Recipe), false negatives represented tokens present in the ASA24 description but not predicted by the algorithm, and false positives were flagged as tokens predicted by the algorithm but absent from the ASA24 description. The Kruskal–Wallis rank sum test with Dunn’s post hoc test was used to evaluate differences in median F1 scores followed by Benjamini–Hochberg multiple testing correction.

Associations between nutrients estimated from ASA24 and Bitesnap were tested using multiple regression and Spearman’s rank partial correlations corrected for age, BMI, ethnicity, and education level. Age and BMI were treated as continuous variables, whereas ethnicity and education level were represented as categorical variables; ethnicity was split into either White or Non-White given the imbalanced representation of Non-Caucasians in this study; education level represented a four-factor variable with the following levels: high school graduate, bachelor’s degree, some college or associate degree, and professional degree (MS, MD, DDS, JD, PhD, or EdD). These covariates were selected on the basis that nutrient intakes co-vary with these demographic variables. Linear models were created using either the stats package or the RVAideMemoire package version 0.9-81-2, in the case of Spearman’s rank partial correlations, with R version 4.1.0. Bootstrapped data from the correlation matrix using 1000 iterations were used to construct 95% confidence intervals. Normality was assessed using the Shapiro–Wilk test on residuals of the model and observing deviations in the residuals of quantile–quantile plots. For non-normally distributed data, transformations were used to approximate normal distributions; otherwise, Spearman’s rank correlations were used.

### 2.8. Text Parsing of Food Descriptions

The textual representations of food descriptions for each meal image were parsed prior to calculating F1 scores for tokens between SNAPMe and Im2Recipe [10] or Facebook (FB) Inverse Cooking [12] datasets. Joined food descriptions for each meal image were cleaned through removing punctuation, lemmatizing each word into the base form (e.g., changing blueberries to blueberry), and removing “stopwords”, which are words that have no relevance to the text-matching task. A token was considered a stopword and removed if it did not provide information that could be used to identify a specific food. This strategy helped to reconcile the different syntaxes of the USDA Food and Nutrient Database for Dietary Studies (FNDDS)17-18 [41] and Recipe 1M [10], the respective databases linked to SNAPMe and publicly available algorithms (Im2Recipe and FB Inverse Cooking) prior to calculating the accuracy (F1 score) between matching food descriptions. Additionally, manual entries were created for ASA24 food descriptions not fully resolved into ingredients (Supplemental File S1).

### 2.9. Study Approvals

This study was registered on ClinicalTrials.gov (Identifier: NCT05008653) and received approval from the University of California Davis Institutional Review Board.

## 3. Results

### 3.1. Participant Characteristics

**Figure 1:**
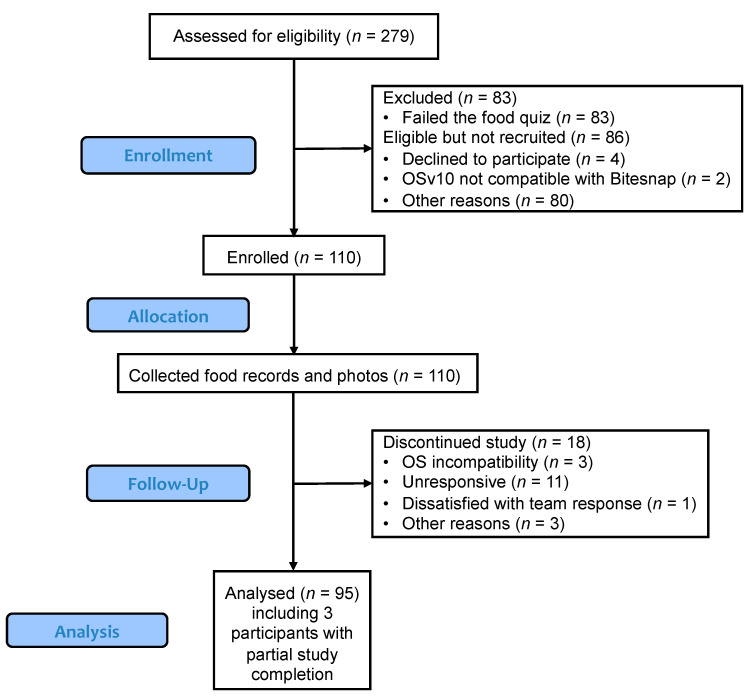
CONSORT diagram depicting the recruitment and enrollment of participants. A total of 95 participants completed the study and were included in the final analysis. CONSORT, Consolidated Standards of Reporting Trials.

The CONSORT diagram (Figure 1) indicates the number of participants who were assessed for eligibility, enrolled, and completed the study and were included in the analysis. A total of 275 study days from 95 participants are included in the database. Participant characteristics, including age, BMI, ethnicity, and gender, are summarized in Table 1. Most of the participants identified as female (*n* = 85), along with 9 males and 1 non-binary individual, and many of the participants were White (58%) or Asian (24%). The mean age was 27.1 years old, and the mean BMI was 23.7.

### 3.2. Database Content and Structure

The SNAPMe database produced by this study is organized in two ways. The “snapme_cs_db” directory is organized for computer scientists, with all photos of a given type in the same sub-directory. The “snapme_nut_db” directory is organized for nutrition scientists, with all data for a given participant and study day in the same sub-directory. The images in “snapme_cs_db” exist as links to the data in “snapme_nut_db.” In other words, there is only one source of data with no copies. The “cs” version of SNAPMe DB additionally contains a master ASA24 linkage file that contains the ASA24 data from all participants alongside names of the associated food photo. The “nut” version of SNAPMe DB contains an ASA24 linkage file within each individual participant and study day directory.

The SNAPMe DB contains four types of food photos in the database: (1) non-packaged food before consumption (“before” photos); (2) non-packaged food after consumption (“after” photos); (3) packaged food, front photo of the package (“front” photos); and (4) packaged food, ingredients label of the package (“ingredient” photos). There are two types of food photos listed in a linkage file, the “before” photos of non-packaged food and the “front” photos of packaged foods, and the type of food photo is indicated in the column “packaged_food” (0 = non-packaged, 1 = packaged). The other two types of filenames are inferred as follows. For “before” photos (non-packaged foods), there are also photos taken “after” consumption with an inferred filename that has the same root name as the “before” photo, but with the added suffix, “_after.jpeg.” For “front” photos (packaged foods), the filename column provides the name of the file, which is the image of the front side of the package. There is also a photo of the ingredients label for the packaged foods and the filename of that photo is the same root name as the front side image but with the added suffix, “_ingredients.jpeg”.

In total, the SNAPMe DB contains 3311 unique food photos linked with 275 ASA24 food records from 95 participants who photographed all foods consumed and recorded food records in parallel for up to 3 study days each for a total of 275 diet days. The SNAPMe DB includes 1475 “before” photos of non-packaged foods, 1436 “after” photos of non-packaged foods, 203 “front” photos of packaged foods, and 196 “ingredient” labels of packaged foods.

### 3.3. Summary Statistics for Food Photos and Food Records in SNAPMe DB

A total of 4761 foods were entered into ASA24 by participants, corresponding to 1153 unique USDA FNDDS FoodCodes. Per study day, there were 17.21 ± 7.94 (mean ± SD) FoodCodes mapped to images. Per image, there were 2.8 ± 2.5 (mean ± SD) FoodCodes with a range of 1 to 17 FoodCodes per image.

When restricting the analysis to only non-packaged foods, there were 4505 “before” foods entered into ASA24, corresponding to 1095 unique FoodCodes. Per study day, there were 16.29 ± 7.78 (mean ± SD) FoodCodes mapped to images of non-packaged foods. Per image of non-packaged foods, there were 3.03 ± 2.58 (mean ± SD) FoodCodes with a range of 1 to 17 FoodCodes per image.

There were 6.17 ± 2.48 (mean ± SD) “Before” or “Front” images taken per study day with a range of 2 to 14 images. Including the companion “After” or “Ingredient” images increased the number of images to 12 per study day, with a range of 4 to 28. Nearly all front/before images were linked to ASA24 records coded as breakfast, lunch, dinner, snack, or beverage. Breakfast was the most common eating occasion, while the beverage category was the least selected occasion (Table 2). This may be because beverages consumed as part of a meal were included as part of that meal occasion.

Based on the ASA24 recalls, there were a small number of entries (36 out of 4761, <1%) without a corresponding image. Seven entries were part of the same meal, and an additional eight entries were toppings or condiments, with a maximum of 21 images that were missing. These included seven beverages, three fruits, three snacks (milk chocolate candy, tortilla chips, and ice cream), and eight other items: soybean curd, beef jerky, lettuce, onion, egg, oats, coconut, and protein powder. These entries are detailed in snapme_cs_db/NAlist.txt in the database download.

### 3.4. Use of SNAPMe DB to Evaluate Ingredient Prediction by the Publicly Available FB Inverse Cooking and Im2Recipe Algorithms

**Figure 2:**
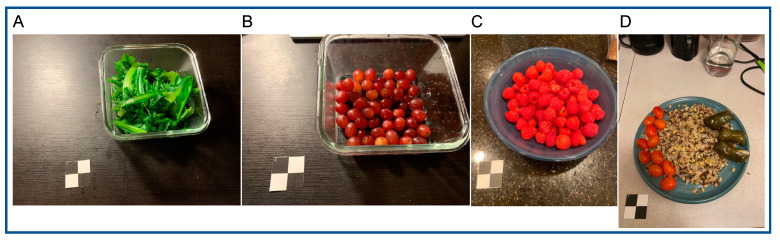
Examples of problematic food photos that could not be processed by BiteSnap representing challenges to food photo-based dietary assessment. (**A**–**C**) Photos with a dark background; (**D**) photo with non-food items.

The FB Inverse Cooking and Im2Recipe algorithms were selected for testing based on their being open source and well documented. The 1477 “before” food photos were processed using the inverse-cooking [12] and Im2Recipe algorithms [10]. Of the 1477 photos, 14 could not be processed without error. The 14 problematic food photos included 13 photos with a dark background (examples in Figure 2A–C) and 1 photo with non-food items in the image (Figure 2D). For the 1463 photos that could be processed, the predicted ingredients were compared with the food items in the ASA24 linkage file. For multi-ingredient foods (e.g., chocolate chip cookie), this proved difficult because the ingredient prediction algorithm appropriately predicted ingredients whereas the ASA24 file contained food items that may or may not have an available recipe from which ingredients can be derived. We therefore further manually “ingredientized” the ASA24 food descriptions when the food item was a mixed dish (Supplemental File S1).

**Figure 3:**
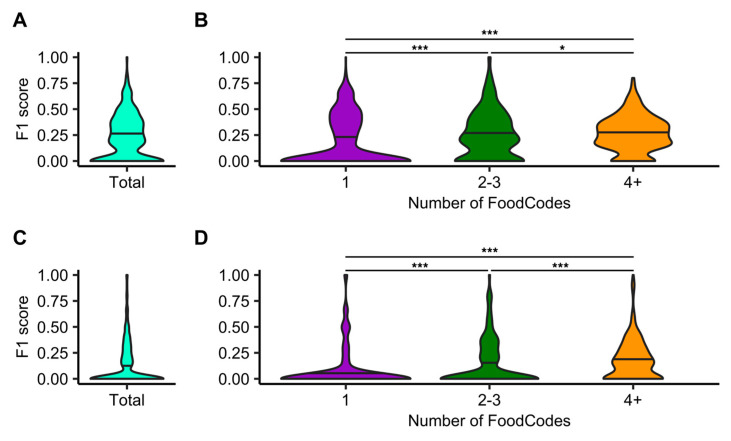
Distributions of F1 scores for predicting ingredients from ASA24 food descriptions using FB Inverse Cooking and Im2Recipe algorithms. (**A**) FB Inverse Cooking F1 scores distributed across the total food photo dataset and (**B**) binned by the number of food codes in a food photo. (**C**) Im2Recipe F1 scores for the total food photo dataset and (**D**) binned by the number of food codes in a meal image. Sample sizes for FoodCode bins: 1 FoodCode, 551; 2–3 FoodCodes, 467; 4+ FoodCodes, 445. Horizontal lines indicate the median values for the Gaussian kernel density estimate. ***, *p* < 0.001, *, *p* < 0.05.

Ingredients predicted by FB Inverse Cooking from ASA24 food records were evaluated using F1 scores. FB Inverse Cooking predictions for individual foods are provided in Supplemental File S2. The mean F1 score for all predictions was 0.23 (Figure 3A). Using the number of food codes from ASA24 records as a proxy for the number of ingredients in an image, the mean F1 score increased successively for images with 2–3 food codes (F1 score = 0.25) and those with 4 or more food codes (F1 score = 0.27) compared to single-food-code images (F1 score = 0.18) (Figure 3B, Dunn’s test: *p* = 0.02). The Im2Recipe algorithm had an overall lower performance with a mean F1 score of 0.13 (Figure 3C; individual predictions in Supplemental File S3). Similar to the FB cooking algorithm, Im2Recipe was better at predicting multi-ingredient foods with a mean F1 score of 0.15 and 0.18 for 2–3 and 4+ food codes, respectively, compared to images corresponding to a single food code (F1 score = 0.08) (Figure 3D; Dunn’s test: *p* = 3 × 10^−9^).

### 3.5. Performance of Bitesnap to Predict Nutrients from Food Photos and User Text Entry

**Figure 4:**
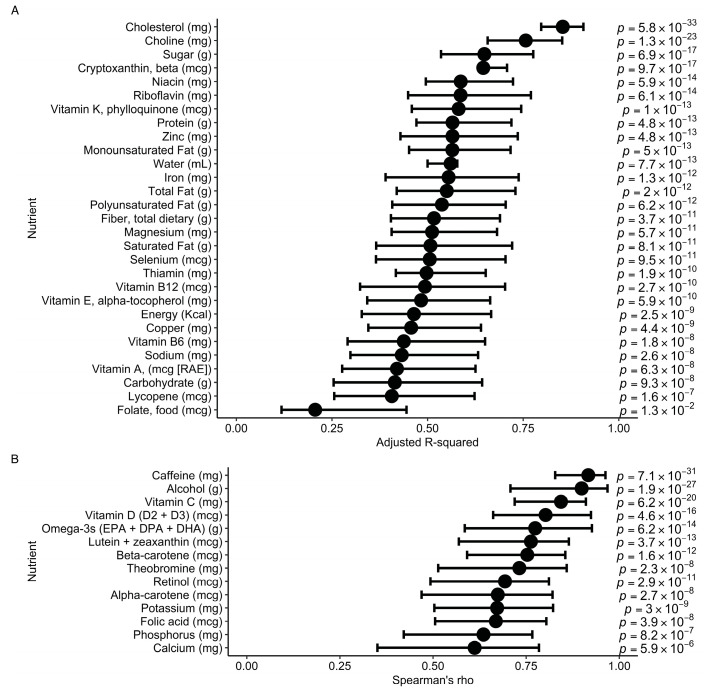
Prediction of nutrient estimates to evaluate the performance of Bitesnap. (**A**) Parametric linear models to assess coefficient of determination between ASA24 and Bitesnap nutrient estimates. (**B**) Spearman’s rank partial correlations between ASA24 and Bitesnap nutrient estimates. Error bars indicate the 95% confidence intervals. Both partial Pearson’s correlation coefficients and adjusted R-squared were adjusted for age, education, ethnicity, and BMI.

As an example of how the benchmark could be used to assess a computer vision approach to nutrient estimation, we evaluated the performance of Bitesnap to predict nutrients from meal images and compared these nutrient estimates with those from each subject’s ASA24 food record. Linear regression was used to assess the strength of correlation while adjusting for the covariates age, BMI, ethnicity, and education level (Figure 4). For the nutrients that met the assumptions of parametric testing, cholesterol was the most predictive (adjusted R^2^= 0.85; 95% CI: 0.81, 0.91; *p* = 5.8 × 10^−33^), and folate from food had the lowest prediction coefficient (adjusted R^2^ = 0.21; 95% CI: 0.11, 0.45; *p* = 1.0 × 10^−2^) (Figure 4A). Non-parametric testing using partial Spearman’s rank correlations revealed caffeine as the best-predicted (Spearman’s rho = 0.92; 95% CI: 0.82, 0.96; *p* = 7.1 × 10^−31^), whereas calcium showed the lowest performance (Spearman’s rho = 0.61; 95% CI: 0.33, 0.78; *p* = 5.9 × 10^−6^).

### 3.6. Participant Experiences Reported in a Post-Study Questionnaire

Participants were asked to reflect on their experiences with a post-study questionnaire (91 of 95 participants completed the questionnaire). Participants indicated a preference for reporting their diet using Bitesnap (67% of participants) compared to ASA24 (33%). When asked to rate their experience with ASA24 and Bitesnap, 90% of participants and 85% of participants, respectively, agreed that ASA24 and Bitesnap accurately captured their diet (ASA24: 18% strongly agreed, 52% agreed, and 20% somewhat agreed; Bitesnap: 19% strongly agreed, 40% agreed, and 26% somewhat agreed). Many participants agreed that both ASA24 (78% of participants) and Bitesnap (80% of participants) were easy to use and not burdensome.

When logging their meal with Bitesnap, 53% of participants reported doing it right after eating so they could remember what they just ate, and 32% of participants reported doing it at the end of the day. For ASA24, which was used in “food record” mode, 73% of participants reported their food intake at the end of the day. On average, however, 77% of participants answered that it took them less time to log meals into Bitesnap than ASA24, 18% of participants answered that it took the same amount of time as ASA24, and 5% answered that it took little more time than ASA24.

For ASA24 and Bitesnap, 27% and 23% of participants, respectively, disliked the lack of multicultural food choices, including Vietnamese, Taiwanese, Italian, Japanese, and Korean food. With the ASA24 system, the participants reported more difficulties reporting homemade food. For recipes that were complex, both platforms were difficult because the participant needed to provide a more detailed description of the meal so that the system could recognize and record what they ate.

## 4. Discussion

In this study, we developed SNAPMe DB, the first publicly available food photo benchmark database collected in the context of dietary assessment by U.S. adults, containing over 3000 food photos linked with ASA24 food records. Previous experience with image-based approaches in clinical studies required the use of trained analysts to refine the annotations after the images were been processed [18]. This dramatically increased the cost and time of clinical studies. A goal for the future would be to maintain data quality while minimizing input from trained analysts. The SNAPMe benchmark can be used to advance this goal through providing a means to compare different methods on the same dataset. SNAPMe DB can be used to evaluate existing models for the prediction of foods, ingredients, and/or nutrients, as captured in the context of “real world” dietary assessment.

As part of our strategy to provide quality data in SNAPMe DB, we screened participants for their ability to judge ingredients and portion sizes. Of the 279 participants screened, 83 (30%) did not pass the screener for the estimation of foods and ingredients, even though our enrollment pool was enriched with students in university nutrition departments. This suggests many participants in the general population will not be able to record food very well and that the use of food photos may improve the dietary assessment of these individuals.

To demonstrate the utility of the SNAPMe DB, we evaluated the ability of publicly available algorithms to predict food ingredients. The Facebook Inverse Cooking and Im2Recipe algorithms resulted in low accuracy. We speculate that the primary reason for this poor performance is a result of these models being trained on recipes, such as the Recipe1M database [10], and not on core or single-ingredient foods (e.g., individual pieces of fruit) or beverages. This observation is supported by a lower mean F1 score for single-FoodCode images compared to images with multiple FoodCodes that would typically represent a mixed meal or recipe. Additionally, images used for training these models were sourced from internet websites, such as cooking and recipe websites, which typically portray the finished dish and may have differences in their appearance compared to a plated dish combined with other foods not part of that recipe. This domain gap will need to be addressed in the development of future models. Differences in syntax between ASA24 food recall output and ingredient prediction algorithms also contributed to lower scores despite parsing and cleaning text descriptions. Methods to standardize text descriptions for food and nutrient databases are needed to harmonize syntactic differences and increase interoperability.

We also evaluated predictions of nutrient estimates using Bitesnap compared with those estimated from ASA24 recalls. Nutrient estimates from Bitesnap modestly correlated with food records, with the lowest performance for food folate (R^2^ = 0.21) and the highest for cholesterol (R^2^ = 0.85). Because cholesterol is derived from animal products, predicting cholesterol content from food photos is simplified based on the limited number of foods that contain cholesterol. Moreover, animal products such as meat are more often consumed in mixed meals rather than single-ingredient foods, facilitating better predictions from the training data. Similarly, alcohol and caffeine were among the top predicted nutrients which occur in a relatively small subset of foods. In contrast, folate from food and calcium both had low predictive values, which may be a result of their ubiquity in the food supply, including many single-ingredient foods. Previous studies evaluating the performance of photo-based dietary assessment methods for nutrient estimation have found correlations of similar strength [42,43,44]; however, those methods shift some of the effort to a dietitian.

In a post-study questionnaire, participants were asked about their experiences. The post-study questionnaire was not ideal for comparing the two systems because we asked participants to do different things for Bitesnap compared to ASA24. For example, participants did not have to record ‘after’ or multiple helpings, or use a sizing marker, in ASA24. Nevertheless, more participants preferred logging food photos than using ASA24.

Participants reported trouble with multicultural foods. For ASA24 and Bitesnap, 23% and 30% of all participants indicated difficulty finding foods in the respective system. ASA24 and Bitesnap both use the USDA’s Food Data Central database, which is limited in recipes or ingredients less commonly used in American diets. Of the 23 Asian participants that completed the post-study questionnaire, 15 reported difficulties logging Chinese, Vietnamese, Thai, Japanese, and Korean foods. As such, multicultural foods need to be added to food composition databases to improve inclusivity and diversity in dietary data methods.

Some recent studies have evaluated imaged-based dietary assessment systems in comparison to weighed food records [44,45] or unweighted food records [46,47]. However, these studies have not released labeled image databases, so they cannot be re-used to compare image processing techniques. In the current study, we elected to use ASA24 food records (which include portion size) rather than weighed food records, as the increased burden of the latter would have likely reduced the number and variety of foods reported.

### Limitations

There are limitations to our study. Restaurant foods were intentionally under-represented as it was expected that participants would not know the details of the ingredients used to prepare these meals. Also, study participants were predominantly young women, so the database does not represent a cross-section of the population. However, it was not intended to represent a cross-section; it was intended to be created by participants with knowledge of food and portion sizes, which is not true of the general population. Finally, our evaluation of existing algorithms was not necessarily comprehensive; these are intended to provide examples of the use of the SNAPMe benchmark.

Importantly, our ground truth data are likely imperfect because we did not provide participants with weighed, packed-out food in coolers. It would have been impractical to prepare thousands of different meals, and those meals would not have been reflective of what or how people eat “in the wild.” In order to represent thousands of meals from diverse participants consuming their usual foods, we implemented safeguards such as (1) the targeted recruitment of participants from nutrition programs, (2) the pre-screening of prospective participants for their ability to identify ingredients and portion sizes, (3) the simultaneous recording of food records, (4) near real-time (within 24 h) checks of food photos and ASA24 records with participants prompted to re-do study days if they recorded foods that did not have an accompanying food photo, and (5) extensive quality control during which each line of each ASA24 food record was linked to a food photo.

## 5. Conclusions

SNAPMe DB is the first food photo benchmark database designed for the comparative evaluation of computer vision methods in dietary assessment for U.S. consumers. The use of food photos to digitize dietary assessment data can be leveraged to reduce diet recall bias and increase the quality of observational data. This study uncovered a domain gap: current ingredient prediction models do not generalize to single-ingredient foods or beverages. The collection of further training photos of single-ingredient images and beverages, retraining models with these images, and re-assessing new models with SNAPMe DB are warranted. While current public algorithms appear to be insufficient, SNAPMe DB will serve as a benchmark for nutrition and computer scientists to reliably evaluate the performance of algorithms developed in the future.

## Figures and Tables

**Table 1:** Demographic characteristics of the study cohort.

	Male	Female	Non-Binary	Total
Participants, *n* (%)	9 (9.5)	85 (89.5)	1 (1.0)	95 (100)
Age, y (Mean ± SD)	31.2 ± 9.36	26.8 ± 6.88	25	27.1 ± 7.17
BMI, kg/m^2^ (Mean ± SD)	23.8 ± 1.7	23.7 ± 4.0	32.1	23.8 ± 3.9
Ethnicity, *n* (%)				
Asian	1 (11.1)	24 (28.2)	0 (0)	25 (26.3)
Black	0 (0)	1 (1.2)	1 (100)	2 (2.1)
Hispanic	0 (0)	3 (3.5)	0 (0)	3 (3.2)
White	6 (66.7)	49 (57.6)	0 (0)	55 (57.9)
White and Other ^1^	2 (22.2)	6 (7.1)	0 (0)	8 (8.4)
Middle Eastern or North African	0 (0)	2 (2.4)	0 (0)	2 (2.1)
Education, *n* (%)				
High school graduate	1 (11.1)	9 (10.6)	0 (0)	10 (10.5)
Some college (1–4 years, no degree)	0 (0)	19 (22.4)	0 (0)	19 (20.0)
Associate degree	1 (11.1)	3 (3.5)	1 (100)	5 (5.3)
Bachelor’s degree	4 (44.4)	37 (45.3)	0 (0)	41 (43.1)
Professional degree ^2^	2 (22.2)	7 (8.2)	0 (0)	9 (9.5)

^1^ Participants reporting White and another ethnicity. ^2^ Professional degrees include an MS, MD, DDS, JD, PhD, or EdD.

**Table 2:** Food photos by eating occasion.

Eating Occasion	Before/Front Images	Images per Study Day (Mean ± SD)
Breakfast	536	1.95 ± 0.99
Lunch	363	1.49 ± 0.79
Dinner	370	1.41 ± 0.69
Snack	321	2.01 ± 1.22
Just Beverages ^1^	110	1.57 ± 1.12

^1^ Beverages that were consumed as part of a meal/snack were included with that eating occasion.

## Data Availability

Data described in the manuscript are now publicly and freely available on Ag Data Commons. The analytic code for reproducing the results can be found at https://github.com/JulesLarke-USDA/SNAPMe (accessed on 16 October 2023).

## Supplementary Materials

The following supporting information can be downloaded at: https://www.mdpi.com/article/10.3390/nu15234972/s1, Supplemental Figure S1. Example question for the food matching test asking participants to identify the ingredients shown in the picture of a sandwich. Supplemental File S1. Examples of manual entries created for ASA24 food descriptions not fully resolved into ingredients, such as mixed dishes. Supplemental File S2. FB Inverse Cooking predictions for individual foods. Supplemental File S3. Individual predictions of F1 score mean.
