# Supplementary material for: Surveying Nutrient Assessment with Photographs of Meals (SNAPMe): A Benchmark Dataset of Food Photos for Dietary Assessment
Source: Nutrients. 2023 Nov 30;15(23):4972. doi: 10.3390/nu15234972 (PMC10708545; doi:10.3390/nu15234972)
Supplement: Supplementary file 1 [file nutrients-15-04972-s001.zip › Supplemental figure S1.pdf]

Supplemental material

Surveying Nutrient Assessment with Photographs of Meals (SNAPMe): A Benchmark Dataset of Food Photos for Dietary Assessment

Jules A. Larke [1], Elizabeth L. Chin [1], Yasmine Y. Bouzid [2], Tu Nguyen [1], Yael Vainberg [2], Dong Hee Lee [3], Hamed Pirsiavash [3], Jennifer T. Smilowitz [2] and Danielle G. Lemay [1,2,*]

[1] United States Department of Agriculture, Agricultural Research Service, Western Human Nutrition Research Center, Davis, CA 95616, USA
[2] Department of Nutrition, University of California Davis, Davis, CA 95616, USA
[3] Department of Computer Science, University of California Davis, Davis, CA 95616, USA; hpirsiav@ucdavis.edu (H.P.)
* Correspondence: danielle.lemay@usda.gov

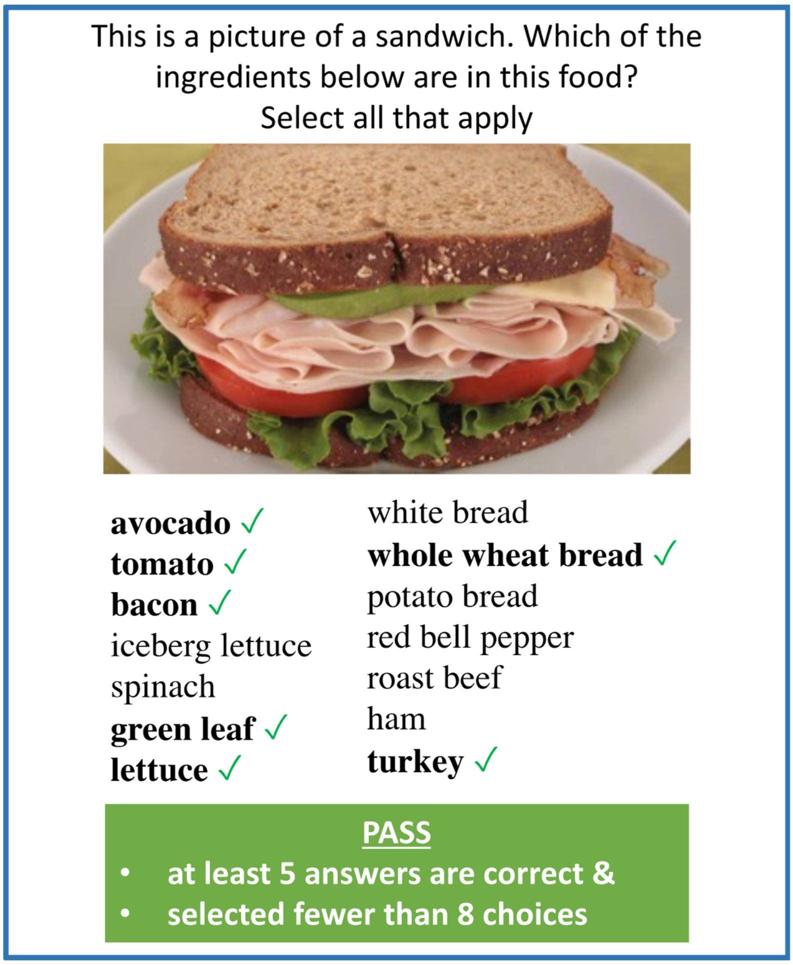

Supplemental Figure S1. Example question for the food matching test asking participants to identify the ingredients shown in the picture of a sandwich.
